# Ponatinib Inhibits Proliferation and Induces Apoptosis of Liver Cancer Cells, but Its Efficacy Is Compromised by Its Activation on PDK1/Akt/mTOR Signaling

**DOI:** 10.3390/molecules24071363

**Published:** 2019-04-07

**Authors:** Chang Liu, Xiuli Mu, Xuan Wang, Chan Zhang, Lina Zhang, Baofeng Yu, Gongqin Sun

**Affiliations:** 1 Department of Biochemistry and Molecular Biology, Shanxi Medical University, Taiyuan 030001, Shanxi, China; xiaophailch@163.com (C.L.); mu13333499415@163.com (X.M.); airourouxuan@163.com (X.W.); qlgcj1120@sina.com (C.Z.); 18335183328@163.com (L.Z.); 2 Department of Cell and Molecular Biology, University of Rhode Island, Kingston, RI 02881, USA

**Keywords:** ponatinib, proliferation, apoptosis, liver cancer cells, PDK1/Akt/mTOR signaling

## Abstract

Ponatinib is a multi-target protein tyrosine kinase inhibitor, and its effects on hepatocellular carcinoma cells have not been previously explored. In the present study, we investigated its effects on hepatocellular carcinoma cell growth and the underlying mechanisms. Toward SK-Hep-1 and SNU-423 cells, ponatinib induces apoptosis by upregulation of cleaved caspase-3 and -7 and promotes cell cycle arrest in the G1 phase by inhibiting CDK4/6/CyclinD1 complex and phosphorylation of retinoblastoma protein. It inhibits the growth-stimulating mitogen-activated protein (MAP) kinase pathway, the phosphorylation of Src on both negative and positive regulation sites, and Jak2 and Stat3 phosphorylation. Surprisingly, it also activates the PDK1, the protein kinase B (Akt), and the mechanistic target of rapamycin (mTOR) signaling pathway. Blocking mTOR signaling strongly sensitizes cells to inhibition by ponatinib and makes ponatinib a much more potent inhibitor of hepatocellular carcinoma cell proliferation. These findings demonstrate that ponatinib exerts both positive and negative effects on hepatocellular cell proliferation, and eliminating its growth-stimulating effects by drug combination or potentially by chemical medication can significantly improve its efficacy as an anti-cancer drug.

## 1. Introduction

Hepatocellular carcinoma (HCC) is a major cause of morbidity and mortality worldwide [1]. Despite rapid progress in liver transplantation, resection, radiofrequency ablation, and chemoembolization, only one third of patients with HCC are suitable for these treatments [2]. HCC is also one of the most chemo-resistant tumors because little survival benefits or tumor responses have been received with many agents [3]. Three small molecule kinase inhibitors, sorafenib, regorafenib, and lenvatinib, have been approved as targeted therapy for advanced stages of HCC for their effects in increasing survival and delaying tumor progression [4,5,6]. All these drugs work mainly as vascular endothelial growth factor receptor (VEGFR) inhibitors to block angiogenesis. However, their use was restricted due to the drug resistance and intolerance. Furthermore, in the past few years, multiple clinical trials with drugs targeting the fibroblast growth factor receptor (FGFR), the VEGFR, the platelet-derived growth factor receptor (PDGFR), the epidermal growth factor receptor (EGFR), the insulin-like growth factor 1 receptor (IGF-1R), the mechanistic target of rapamycin (mTOR), and the transforming growth beta (TGFβ) signaling pathways have been conducted, but none have been shown to be effective in improving patient survival [7]. The molecular pathogenic mechanism of HCC is very complicated, involving up-regulation of Ras/Raf/Mek/Erk and phosphatidylinositol 3-kinase (PI3K)/Akt/mTOR signaling transduction pathways and aberrant expression of receptor tyrosine kinases [4]. For any signaling inhibitor to be developed into an anticancer drug, it is critical to understand its molecular mechanisms of action and its effects on oncogenic processes and normal physiological processes.

Several cell signaling pathways are involved in sustaining cancer cell proliferation, and identifying specific pathways for a given cancer enables us to design targeted treatments [8]. The mitogen-activated protein kinase (MAPK) pathway and the pPI3K/Akt/mTOR pathway have emerged as crucial signaling cascades involved in regulating cell growth, proliferation, and cell death in various cancers [9]. Protein kinase B (Akt) is essentially an anti-apoptotic protein, which phosphorylates various protein targets controlling cell proliferation, survival and motility [6]. In addition, dysregulated Stat3 signaling is also involved in tumorigenesis by affecting cell growth, angiogenesis and prevention of apoptosis [10,11]. 

Receptor and cytosolic protein tyrosine kinases (PTKs) also play crucial roles in driving proliferation of various cancers. Several rPTKs are critical to HCC development, progression and metastasis, including EGFR, VEGFR, FGFR, PDGFR, and c-mesenchymal-epithelial transition factor-1 (c-Met) [12,13,14]. Aberrant Src activity triggers dysregulation of numerous cellular processes including proliferation, apoptosis, invasion, migration, and angiogenesis, suggesting Src and Src family kinases play central roles in tumor progression and metastasis [15]. The association between HCC and Src expression and activation is well established, and activated c-Src may be responsible for malignant transformation of hepatocytes [16].

Ponatinib (AP24534), an orally available and potent multi-targeted kinase inhibitor, has been approved by the Food and Drug Administration (FDA) to treat imatinib-resistant patients with chronic myelogenous leukemia [17,18]. Ponatinib inhibits both native and mutant forms of BCR-ABL, including the T315I gatekeeper mutant that is resistant to other tyrosine-kinase inhibitors (TKIs) [19,20]. Besides its activity against BCR-ABL, ponatinib also inhibits approximately 50 other PTKs with IC_50_ values below 100 nM, including many kinases in the PDGFR, Src, VEGFR, Eph receptor (EphR), and FGFR families [20,21]. In a panel of 14 cell lines, including breast, lung, and colon cancer cell lines, ponatinib inhibited FGFR-mediated signaling and cell growth significantly in a variety of cell lines [22]. Ponatinib also inhibited the migration and the invasion of breast and orthotopic neuroblastoma cancer cells in vitro and in mouse models, and the migration and mammosphere formation of breast cancer cells in vitro and arrested breast cancer lung metastasis in mouse models [23,24]. 

To date, the effects of ponatinib on HCC have not been determined. In the present study, we examined the effects of ponatinib on the proliferation, cell cycle, and apoptosis of HCC cells, and determined its effects on the cell signaling network. This study sheds light on the complicated molecular mechanisms by which a multi-targeted TKI such as ponatinib can affect the biochemistry and physiology of targeted cells and reveals approaches that can improve its efficacy. The information provides a basis for further laboratory research and development of ponatinib as an anti-cancer drug.

## 2. Results

### 2.1. Ponatinib Potently Inhibits Viability and Proliferation of HCC Cell Lines

**Figure 1:**
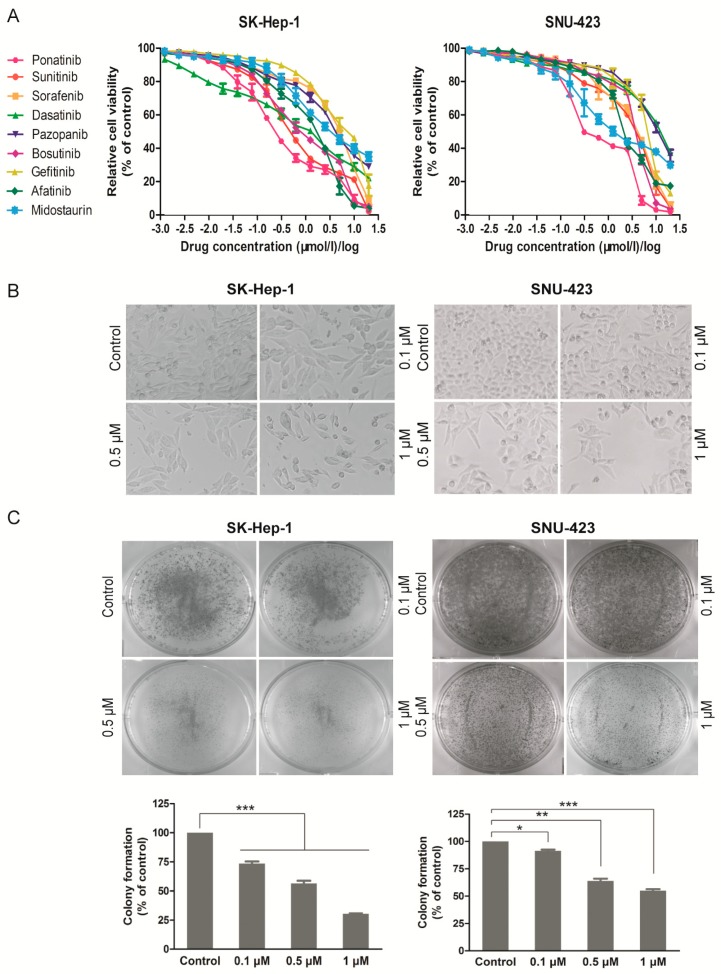
Efficacy of individual PTK inhibitors against different hepatocellular carcinoma (HCC) cell lines and cytotoxicity of ponatinib. (**A**) Cell proliferation was examined by 3-(4,5-dimethyl-2-thiazolyl)-2,5-diphenyl-2-*H*-tetrazolium bromide (MTT) assay. HCC cell lines including SK-Hep-1 and SNU-423 cells were seeded in 96-well plates and then treated with different concentrations of drugs for 72 h. The relative cell viability was calculated and shown. (**B**) Cell proliferation inhibition was observed by microscopy (×100). SK-Hep-1 and SNU-423 cells were plated in 24-well plates and exposed to indicated doses of ponatinib for 36 h. (**C**) SK-Hep-1 and SNU-423 cells were plated in six-well plates (500 cells/well) and treated with various concentrations of ponatinib for 10 days, then the clonogenic ability was measured and shown. The bars indicate the colony formation ability of different groups. Each experiment was carried out three times. (*n* = 3; *, *p* < 0.05; **, *p* < 0.01; ***, *p* < 0.001).

The 3-(4,5-dimethyl-2-thiazolyl)-2,5-diphenyl-2-H-tetrazolium bromide (MTT) assay was performed on SK-Hep-1 and SNU-423 cells treated with increasing concentrations of the nine TKIs (Table 1) for 72 h, and the dose response curves are shown in Figure 1A, and the IC_50_ values are listed in Table 2. The most potent inhibitor was ponatinib, which inhibited the viability of the two cell lines with IC_50_ of 0.288 ± 0.044 μM (SK-Hep-1) and 0.553 ± 0.041 μM (SNU-423), respectively. Ponatinib treatment resulted in decreased cell numbers and irregular morphology (Figure 1B) and inhibited colony formation of SK-Hep-1 and SNU-423 cells at sub-μM concentrations (Figure 1C). Collectively, these results indicate that ponatinib potently inhibits the viability and proliferation of these HCC cell lines. 

### 2.2. Ponatinib Induces Apoptosis in HCC Cells

**Figure 2:**
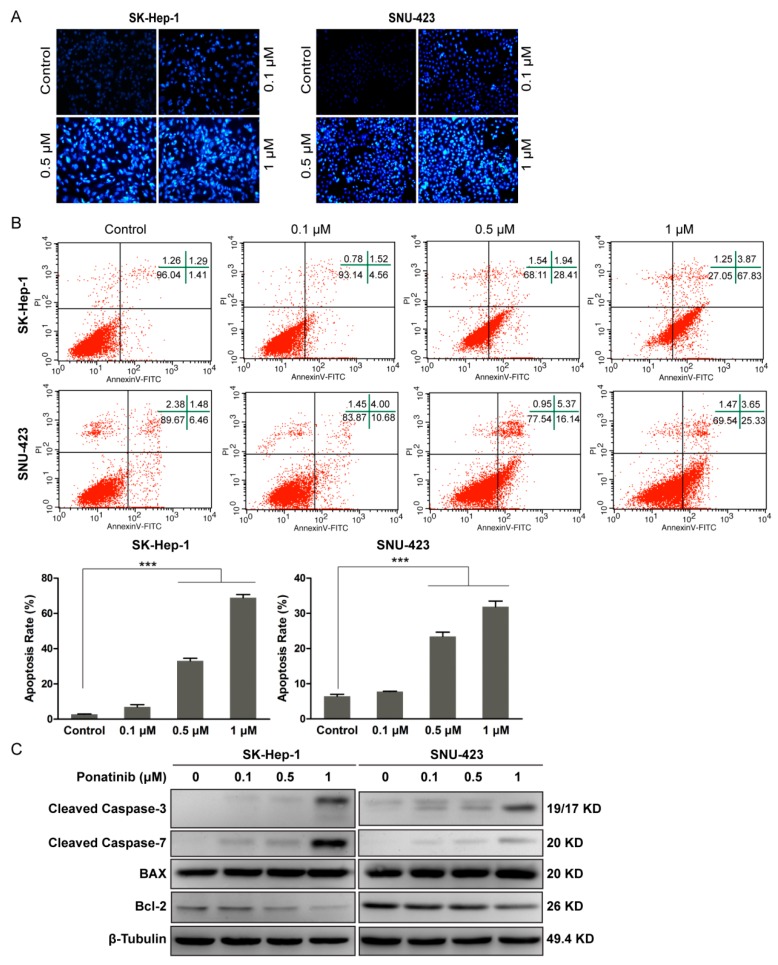
Ponatinib induced cell apoptosis in SK-Hep-1 and SNU-423 cells in a concentration-dependent manner. (**A**) SK-Hep-1 and SNU-423 cells were stained with Hoechst 33342 observed on fluorescence microscopy (×200). (**B**) Flow cytometry analysis of apoptosis (data from three independent experiments). (**C**) Western blot analysis of cleaved caspase-3, cleaved caspase-7, Bax, Bcl-2, and β-Tubulin (control) in treated cells.

We determined whether the decrease in cell viability could be attributed to the induction of apoptosis. SK-Hep-1 and SNU-423 cells were treated with ponatinib for 36 h and stained with Hoechst 33342. As shown in Figure 2A, ponatinib induced apoptosis in the two cell lines in a dose-dependent manner. Nuclear condensation with bright-blue fluorescence and cellular fragmentation were observed even at 0.1 μM ponatinib.

Cell apoptosis was measured by flow cytometry using the Annecin V-FITC/PI apoptosis detection kit. After treatment with ponatinib for 36 h, the combined early (lower right quadrant) and late (upper right quadrant) apoptosis rates in Figure 2B increased with increasing ponatinib concentration. The apoptosis rate of SK-Hep-1 cells increased from 2.0 ± 0.7% (control) to 7.0 ± 0.92%, 33.1 ± 0.75%, and 68.9 ± 2.8% when incubated with 0.1, 0.5, and 1 μM ponatinib, respectively. Similarly, that of SNU-423 cells increased from 6.5 ± 1.44% (control) to 7.8 ± 6.88%, 23.5 ± 1.99%, and 31.9 ± 2.92% by the same treatments.

Previous reports have demonstrated that ponatinib induces apoptosis in tumor cells through a caspase 3- and 7-dependent mechanism [22,25,26,27]. As shown in Figure 2C, ponatinib treatment promoted cleavage of caspase-3 and -7 in SK-Hep-1 and SNU-423 cells even at 0.1 μM and more prominently at 1 μM. It was reported previously that caspase-3 and -7 are not only involved in the execution phase of apoptosis but also regulate events in apoptosis initiation, such as Bax translocation and cytochrome c release [28,29]. As shown in Figure 2C, ponatinib treatment of SK-Hep-1 and SNU-423 cells downregulated Bcl-2 and upregulated Bax in a concentration-dependent manner, indicating that ponatinib-induced apoptosis is associated with the mitochondrial apoptotic pathway.

### 2.3. Ponatinib Causes Cell Cycle G1 Phase Arrest in HCC Cells

**Figure 3:**
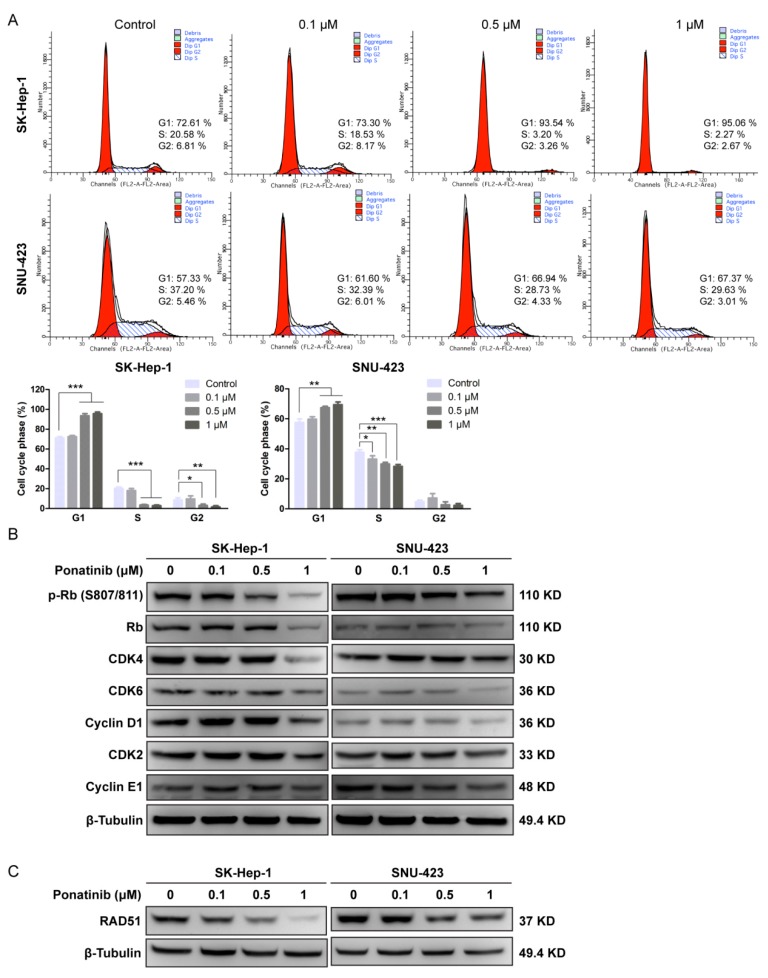
Effect of Ponatinib on the percentage of cells in the G1 phase and the expression of G1 phase-related proteins. (**A**) SK-Hep-1 and SNU-423 cells were treated with ponatinib, and cell cycle distribution was analyzed by flow cytometry. The percentages of cells in cell cycle phases were calculated from three independent experiments. (**B**) Western blot analysis of Rb/p-Rb, CDK4, CDK6, Cyclin D1, CDK2, Cyclin E1, and β-Tubulin (control) in the SK-Hep-1 and SNU-423 cells treated with ponatinib. (**C**) The effect of ponatinib on the concentration of Rad51 in SK-Hep-1 and SNU-423 cells.

To investigate how the cell cycle is affected, ponatinib-treated SK-Hep-1 and SNU-423 cells were analyzed by flow cytometry (Figure 3A). The population of cells in the G1 phase increased with increasing concentrations of ponatinib for both cell lines, suggesting that the drug blocked the progression from G1 to S phase. Western blotting analysis (Figure 3B) indicated that the drug at 1 μM resulted in decreased levels of Rb, pRb, CDK4, CDK6, and Cyclin D1, while CDK2 and Cyclin E1 levels were not affected. Because both CDK4/6/Cyclin D1 and CDK2/Cyclin E1 complexes are capable of phosphorylating Rb and regulating G1 to S transition, these results suggest that ponatinib blocks G1 to S transition by reducing CDK4/6/Cyclin D1 function.

Some PTK inhibitors such as gefitinib and crizotinib can cause DNA damage [30,31] and result in increased expression of RAD51, a DNA repair protein in the homologous recombination repair pathway [32,33]. We evaluated the expression level of RAD51 in response to ponatinib treatment (Figure 3C). The RAD51 expression level decreased significantly with ponatinib treatment above 0.5 μM in both SK-Hep-1 and SNU-423, suggesting that the homologous recombination pathway was activated. Whether this effect was because ponatinib interfered with the homologous recombination repair signaling or actually resulted in DNA damage was not clear.

### 2.4. Ponatinib Interferes with Multiple Cell Signaling Pathways in the HCC Cells

The above results demonstrated that ponatinib inhibited cell proliferation, induced apoptosis, caused cell cycle arrest at G1 phase, and caused DNA damage or interfered with the DNA damage repair response. We next investigated the activation status of some key PTK-mediated signaling pathways.

**Figure 4:**
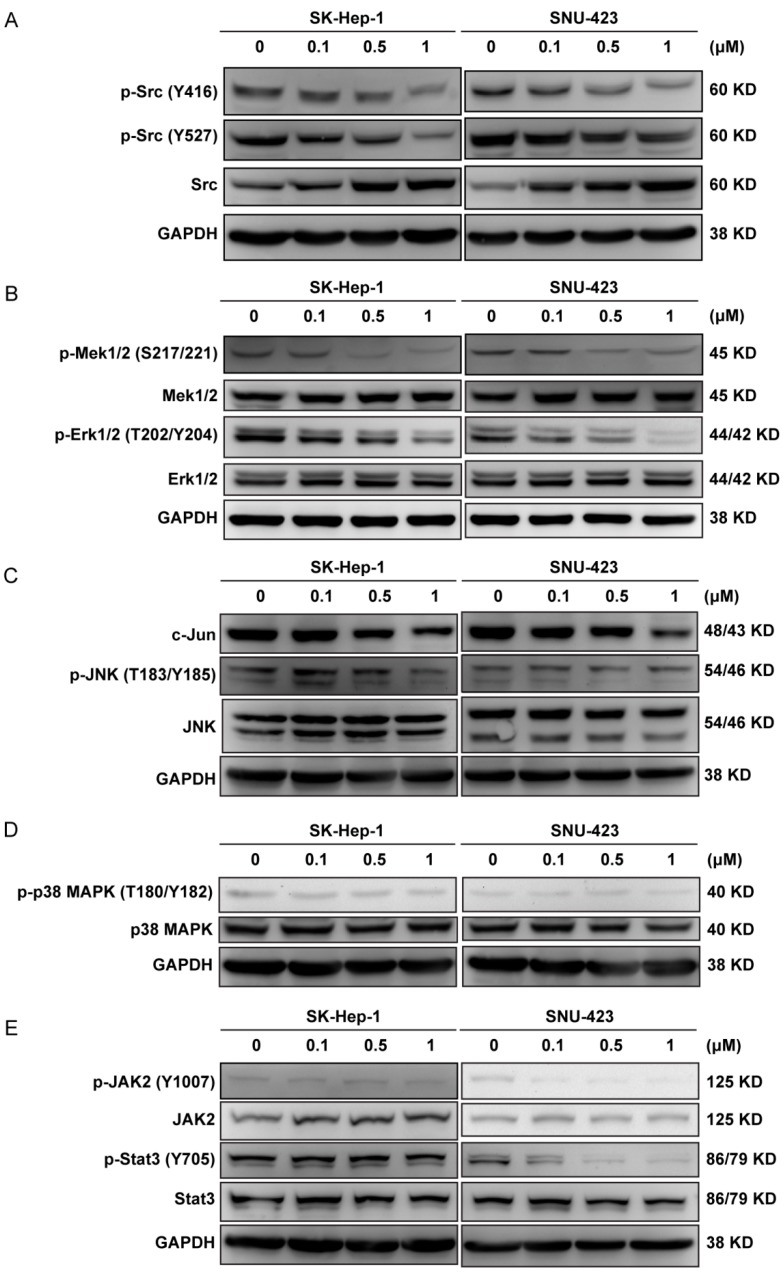
Effect of ponatinib on the phosphorylation of signaling proteins. (**A**) Effect of ponatinib on the phosphorylation of Src on Tyr416 and Tyr527. (**B**–**E**) Effect of ponatinib on the phosphorylation of Mek, Erk, JNK, c-Jun, p38, Jak2, and Stat3. Glyceraldehyde-3-phosphate dehydrogenase (GAPDH) was used as a control.

The soluble PTK Src is a well-established oncoprotein contributing to the proliferation of many types of cancer cells. Ponatinb inhibits Src with an IC_50_ of 5.4 nM [17]. As shown in Figure 4A, ponatinib caused an increase in Src expression and a decrease in Src phosphorylation on both Tyr416 and Tyr527 residues in a dose-dependent manner in SK-Hep-1 and SNU-423 cells. Because Src was activated by autophosphorylation on Tyr416, decreased phosphorylation on Tyr416 indicated that Src kinase activity was inhibited by ponatinib. Src was inactivated by the phosphorylation on Tyr527, which was catalyzed by the C-terminal Src kinase (Csk) [34]. The decreased phosphorylation on Tyr527 indicated that Csk was also inhibited by ponatinib. Indeed, ponatinib inhibited Csk with an IC_50_ of 12.7 nM [17]. The net effect of ponatinib on Src kinase activity was likely a potent inhibition, because even Src that was unphosphorylated on Tyr527 would still have been inhibited by ponatinib. Because Src kinase is a key mediator of cell proliferation, inhibition of Src was likely one reason ponatinib inhibited the proliferation of these cells.

The MAPK pathway is activated by numerous extracellular signals and regulates cell proliferation, differentiation, and death. The MAP kinases include ERKs/MEKs, JNKs, and p38/SAPKs families [35], and ERKs/MEKs are involved in growth regulation. Because ERKs/MEKs are activated by rPTKs, if ponatinib inhibits any rPTKs, it would cause a down regulation of ERKs and MEKs. As shown in Figure 4B, Erk1/2 and MEK1/2 expression level did not change, but their phosphorylation levels decreased by ponatinib treatment, indicating that the MAPK pathway was inactivated by ponatinib. This was likely due to ponatinib inhibiting upstream rPTKs. More than 25 rPTKs in the EPH, FGFR, PDGFR, RET, TRK, and VEGFR families were sensitive to ponatinib with IC_50_ values below 50 nM [17,18]. In contrast, the expression and phosphorylation levels of JNK, c-Jun, and p38 MAPK were not affected by ponatinib (Figure 4C,D). These results indicated that the growth-stimulating ERK/MEK MAPK pathway was selectively down regulated by ponatinib, while the JNK/c-Jun and the p38 MAP kinase pathways, most commonly involved in mediating stress and cytokine responses, were not affected.

Inhibition of Bcr-Abl was reported to result in downregulation of STAT3 via JAK and MEK pathways [36], and ponatinib has been reported to inhibit phosphorylation of JAK2 both in BaF3/T315I cells in vitro and in leukemia models in vivo [37]. Therefore, we analyzed if the JAK2/STAT3 pathway was affected by ponatinib (Figure 4E). Ponatinib did not affect the expression levels of JAK2 and STAT3, nor did it affect the phosphorylation levels of p-JAK2 or p-STAT3 in SK-Hep-1 cells. In SNU-423 cells, however, ponatinib potently inhibited the activation of the JAK2 (p-JAK2) and the phosphorylation of STAT3 (p-STAT3) without affecting their protein levels. The effect of ponatinib on JAK2/STAT3 phosphorylation became clear even at 0.1 μM. The cell specificity of SK-Hep-1 versus SNU-423 indicated that the ponatinib effect on JAK2/STAT3 pathway was cell background-dependent.

### 2.5. Ponatinib Activates the Akt/mTOR Pathway, and Blocking Akt/mTOR Signaling Sensitizes HCC Cells to Ponatinib Inhibition

**Figure 5:**
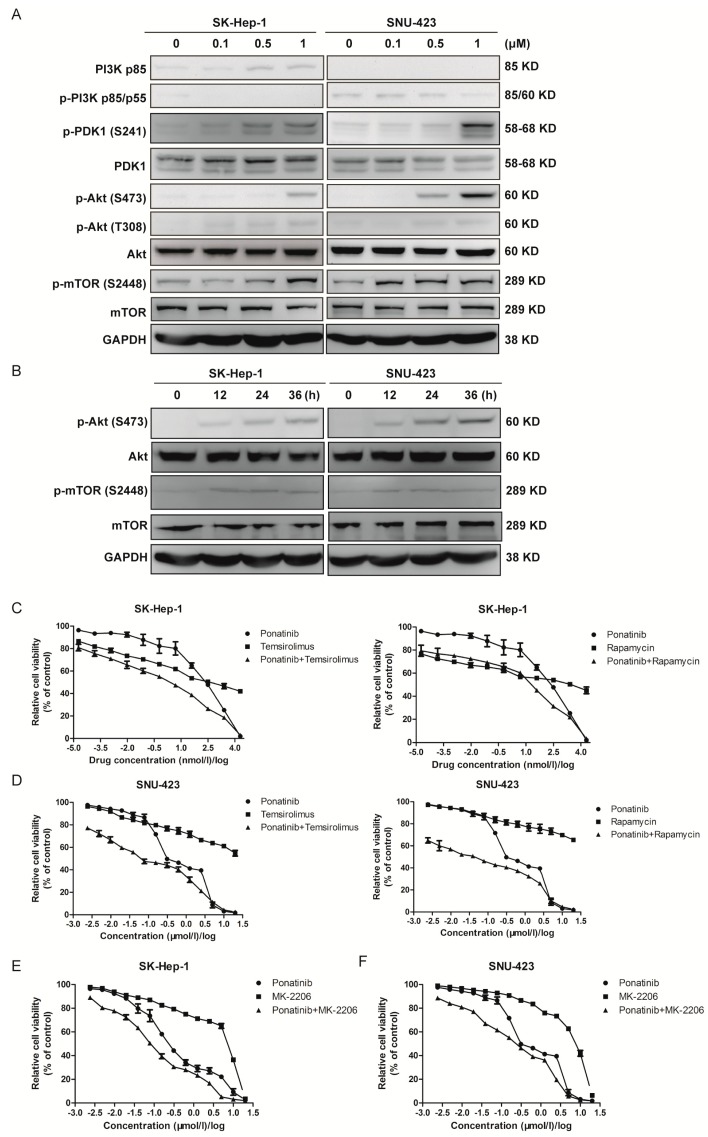
Effect of ponatinib on the protein kinase B (Akt)/mechanistic target of rapamycin (mTOR) signaling pathway. (**A**) Effects of ponatinib on the protein concentration and phosphorylation of PI 3-kinase, PDK1, Akt, and mTOR. (**B**) Time course of ponatinib (1 μM) effect on the phosphorylation of Akt and mTOR. (**C**) Effect of ponatinib on SK-Hep-1 cell viability in the presence and absence of mTOR inhibitors, temsirolimus and rapamycin. (**D**) Effect of ponatinib on SNU-423 cell viability in the presence and absence of mTOR inhibitors, temsirolimus and rapamycin. (**E**) Effect of ponatinib on SK-Hep-1 cell viability in the presence and absence of an Akt inhibitor, MK-2206. (**F**) Effect of ponatinib on SNU-423 cell viability in the presence and absence of an Akt inhibitor, MK-2206.

We next investigated the activation status of some key PTK mediated signaling pathways. The activation of the PI3K/Akt/mTOR signaling pathway is a crucial event in the progression of human liver cancer [38]. Many PTK inhibitors block cancer cell proliferation by inhibiting the Akt/mTOR signaling pathway [39,40,41]. We determined if ponatinib inhibited the HCC cell proliferation by blocking this pathway (Figure 5A). Both PI3K and p-PI3K levels were low but consistently detectable in both SK-Hep-1 and SNU-423 cells. PDK1 and Akt protein levels did not change upon ponatinib treatment, but p-PDK1, p-Akt (S473), and p-Akt (T308) levels increased by ponatinib treatment. The phosphorylation of both Ser473 and Thr308 was responsible for fully activating Akt [40]. These results indicated that ponatinib activated PDK1 and Akt in these cells. Activated Akt phosphorylated mTOR on Ser2448 to positively regulate protein translation [42]. The p-mTOR (S2448) level also increased with ponatinib treatment (Figure 5A). These results suggested that ponatinib treatment activated the Akt-mTOR pathway. Time course experiments showed that activation of Akt was time-dependent in the 36 h treatment period (Figure 5B). These observations were consistent with the observations in pancreatic ductal adenocarcinoma cells, where ponatinib also increased activation of Akt [41].

Because ponatinib activates the Akt/mTOR pathway, and the activation of this pathway is expected to give the cells a survival advantage, we wondered if activation of the Akt/mTOR pathway compromised ponatinib as a drug against the HCC cells. To test this possibility, we determined if inhibition of mTOR signaling with mTOR inhibitors rapamycin or temsirolimus would synergistically enhance the potency of ponatinib (Figure 5C,D). SK-Hep-1 had IC_50_ values of 0.288 ± 0.044 μM, 0.341 ± 0.197 μM, and 1.718 ± 0.282 μM for ponatinib, temsirolimus, and rapamycin, respectively (Figure 5C, Table 3), while the IC_50_ values were 0.002 ± 0.001 and 0.019 ± 0.009 μM for the ponatinib+temsirolimus combination (1:1 ratio) and ponatinib+rapamycin combination (1:1 ratio) (Table 3). Similarly, SNU-423 had IC_50_ values of 0.553 ± 0.041 μM, 11.407 ± 0.104 μM, and 22.665 ± 0.097 μM for ponatinib, temsirolimus, and rapamycin, respectively, but the IC_50_ values were 0.066 ± 0.009 and 0.042 ± 0.007 μM for the ponatinib+temsirolimus combination and ponatinib + rapamycin combinations, respectively (Table 3). The combination indexes (CI) at 50% inhibition for both cells were 0.122 or below, indicating strong synergy between ponatinib and the mTOR inhibitors. This result indicated that the combinations of ponatinib and mTOR inhibitors were eight to 77 times more potent than their separate effects. The strong synergy between ponatinib and temsirolimus or rapamycin indicated that blocking the mTOR signaling pathway made ponatinib much more potent as an inhibitor of proliferation in these two cells. Blocking Akt activity with MK-2206 had a similar, although less dramatic, synergistic effect on ponatinib inhibition of SK-Hep-1 and SNU-423 proliferation (Figure 5E,F).

### 2.6. Ponatinib Did Not Affect PTEN Expression Level

**Figure 6:**
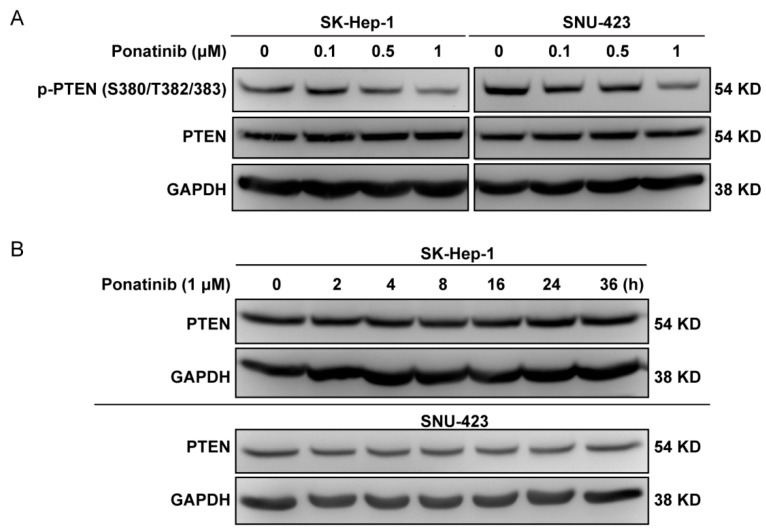
Effect of ponatinib on PTEN. (**A**) Effect of ponatinib on the phosphorylation of PTEN on Ser380/Thr382/383. For the Western blot analysis, SK-Hep-1 and SNU-423 cells were treated with ponatinib at indicated concentrations for 36 h. (**B**) Time course of ponatinib (1 μM) effect on PTEN in SK-Hep-1 and SNU-423 cells.

There is an established signaling mechanism that can potentially explain how ponatinib can activate PDK1 and Akt. PDK1 and Akt are activated by phosphatidylinositol 3,4,5-trisphosphate (PIP_3_), and the accumulation of PIP_3_ is controlled by the balance between phosphatidylinositol 3-kinase and the PTEN phosphatidylinositol 3-phosphatase. Because PI 3-kinase is not affected by ponatinib, PTEN becomes a plausible candidate for the ponatinib effect. It is established that the PTK Rak/Frk phosphorylates PTEN on Tyr336 and prevents PTEN degradation by the ubiquitin-mediated degradation of PTEN [43]. It is also known that ponatinib inhibits Rak/Frk with an IC_50_ of 1.3 nM. Thus, it is logical that ponatinib would inhibit Rak/Frk activity, which results in PTEN hypophosphorylation on Tyr336 and PTEN degradation. The loss of PTEN would lead to the accumulation of PIP_3_, which activates PDK1 and Akt. Therefore, we analyzed if PTEN level was affected by ponatinib (Figure 6A,B). Ponatinib treatment did not have any effect on the expression level of PTEN and might have inhibited the phosphorylation of PTEN on Ser/Thr residues in SK-Hep-1 and SNU-423 cells. Time course experiments showed that PTEN level did not change in the 36 h treatment period (Figure 6B). Taken together, our results suggested that ponatinib did not cause PTEN degradation. It was not clear how ponatinib activated PDK1 and Akt.

## 3. Discussion

Sorafenib, a multiple target tyrosine kinase inhibitor like ponatinib that targets PDGFR, VEGFR, and Raf kinases, is also one of the FDA-approved targeted therapies for advanced HCC for its antiproliferative and antiangiogenic effects [4]. However, drug intolerance and resistance to sorafenib restricted its use after several negative phase III clinical trials. Thus, it is imperative to develop more powerful kinase inhibitors that can effectively block HCC proliferation.

The original objective of this work was to determine what signaling drugs the HCC cell lines would be sensitive to and then use that information to elucidate the signaling mechanisms that drive the proliferation of these cells and to develop a strategy to treat such cancer cells. The initial screening revealed that ponatinib was the most potent inhibitor for the HCC cell lines. Characterization of the HCC cell response to ponatinib revealed that ponatinib had a complex and wide spread effect on the HCC cells. Some of the effects were consistent with the objective of blocking HCC cell proliferation, and others may have been counterproductive toward that objective. Elucidating the complex interactions between a drug and the complex signaling network of the target cells is essential in understanding the efficacy or the ineffectiveness of a drug and provides a basis for designing a more effective drug.

The overall objective of the current study was to understand the complicated effects of ponatinib on HCC cell lines SK-Hep-1 and SNU-423, which might be the foundation for developing an effective treatment. With this purpose, we first explored the response of two HCC cell lines to nine tyrosine kinase inhibitors and found ponatinib displayed the best inhibition. Then, we investigated the effects of ponatinib on the proliferation of SK-Hep-1 and SNU-423 cells. Our results showed that ponatinib caused apoptosis in SK-Hep-1 and SNU-423 cells, which was confirmed by the upregulation of caspase-3/7 activity. The cells accumulated in the G1 phase in response to the treatment, which was apparently caused by inhibiting the CDK4/6/Cyclin D1 complex. Moreover, the inhibition of the cell cycle progression seemed to be due to the inhibition of the ERK pathway, as Mek 1/2 and Erk 1/2 phosphorylation level all decreased. We also observed activation of the Akt/mTOR pathway in both cells and inhibition of Jak2/Stat3 only in SNU-423 cells.

Many potent inhibitors have been developed against well-established oncogenic kinases, yet such inhibitors often fail in clinical trials as effective drugs. The reasons are likely multiple and complex, and understanding the reasons behind the failures holds the key to developing more effective targeted therapy. The findings in the current study provide an example for one such reason. Ponatinib is a potent multi-targeted PTK inhibitor and is a very useful and critical agent for chronic myeloid leukemia (CML) and philadelphia chromosome-positive acute lymphoblastic leukemia (Ph+ALL), particularly those with T315I mutations. In addition to inducing apoptosis and inhibiting cell division and proliferation by blocking several proliferation-stimulating signaling pathways, it also activates the PDK1/Akt/mTOR survival pathway, which severely compromises ponatinib as an anti-cancer drug.

Ponatinib inhibits nearly half (28 receptor PTKs and 15 soluble PTKs) of all human PTKs (total = 94) with IC_50_ values below 50 nM [17]. Consistent with this broad specificity, ponatinib causes apoptosis and induces cell cycle arrest at the G1 phase in SK-Hep-1 and SNU-423 HCC cells. At the molecular level, apoptotic caspase-3/7 activities are activated, and the CDK4/6/Cyclin D1 complex is inhibited by ponatinib. A number of kinase signaling pathways are inhibited by ponatinib, including the MAPK pathway, the Src signaling pathway, and the JAK2/STAT3 pathway. Inhibition of these pathways is likely contributing to the anti-proliferative effects of ponatinib.

Surprisingly, ponatinib also activates the PDK1/Akt/mTOR signaling pathway. This activation reduces the effectiveness of ponatinib as an inhibitor against HCC proliferation. Even though the mTOR and Akt inhibitors alone are not potent inhibitors of HCC cell proliferation, they dramatically sensitize the cells to killing by ponatinib, indicating that the activation of PDK1/Akt/mTOR likely enhances the ability of the cells to survive. This result suggests that ponatinib would have been a much more potent inhibitor for HCC cells had it not activated the PDK1/Akt/mTOR pathway. There are conflicting reports on if ponatinib inhibits [20,44,45] or activates [41] Akt signaling in the literature. The discrepancy suggests that the effects of ponatinib on Akt signaling may be dependent on cell signaling background.

The findings suggest that the unintended activation of the PDK1/Akt/mTOR may be one reason why a potent PTKI such as ponatinb, despite its potent inhibition of its intended targets, may not work well against some cancers. The results suggest two potential approaches to overcome this problem. In the long term, developing PTK inhibitors that only inhibit the cancer promoting pathways without activating Akt would offer the best hope for effective agents for targeted therapy. For this purpose, elucidating the molecular mechanisms of drug action is essential. In the short term, combination of Akt/mTOR pathway inhibitors with ponatinib can also overcome this obstacle.

In addition to ponatinib, other PTK inhibitors have been reported to activate Akt. Another broad PTKI, dasatinib, has also been shown to activate Akt [46]. Crizotinib, a Met kinase inhibitor, was shown to activate Akt signaling in gastric cancer cells [31], even though it had an overall antiproliferative effect on these cells. Furthermore, co-inhibition of Akt signaling enhanced the anti-proliferative effect of crizotinib on gastric cancer cells, indicating that the activation of the Akt pathway was counterproductive in crizotinib’s antiproliferative effect on gastric cancer cells. U0126, a non-ATP competitive MEK inhibitor, was also shown to trigger significant increase in Akt phosphorylation at both S473 and T308 in neural progenitor cells [47]. Thus, activation of Akt/mTOR signaling may be an undesirable side effect for many PTK inhibitors. How wide spread this phenomenon is awaits further investigation.

The effects of ponatinb on HCC cell signaling are complex but consistent with published data. The inhibition of phosphorylation of Src on Tyr527 is likely due to ponatinib inhibiting Csk (IC_50_ = 12.7 nM) [17]. Even though blocking Tyr527 phosphorylation on Src is supposed to activate Src activity, this activation is likely inconsequential in its overall effect on Src function, because Src activity is also inhibited by ponatinib. Ponatinib also inhibits Jak2 with an IC_50_ less than 200 nM [17].

A broad spectrum PTK inhibitor can be beneficial if multiple PTKs are contributing to a cancer cell’s proliferation, and a multi-PTK inhibitor such as ponatinib can be used to block all the involved PTKs. However, a broad spectrum PTK inhibitor may block other PTKs that may play important normal physiological functions. Inhibiting such PTKs may result in undesirable effects such as activating Akt/mTOR pro-survival signaling or even cellular toxicity. Although we focused on a few cancer-relevant signaling pathways, the effect of ponatinib on other PTKs may also have important impacts on cell physiology, including potential cytotoxic effects. Indeed, there are numerous reports of toxicity caused by ponatinib and other broad spectrum PTK inhibitors. For example, treatment of HepG2 liver cancer cells with ponatinib at low μM concentrations resulted in severe mitochondrial toxicity and diminished ATP production [48]. All approved BCR-ABL inhibitors display some levels of myocyte, cardiovascular and hepatic toxicity, and the toxicity in general correlates to the lack of specificity [49,50,51]. In addition, toxicities concerned with TKIs also include hyperglycemia, pulmonary hypertension, pneumonitis, pleural effusion, musculoskeletal pain, lipase elevation/pancreatitis, and myelosuppression [52,53]. Careful management of the off-target side effects without losing the on-target benefit would be a key to effective development and application of such inhibitors.

In conclusion, our present study indicates that ponatinib can induce cytotoxicity, caspase-dependent cell apoptosis, and cell cycle arrest, which might be a potential agent for liver cancer treatment. The study provides evidence ponatinib inhibits a number of pathways to lead to tumor inhibition. In future studies, gene knockout or silencing of particular pathways will be able to provide further confirmation for such mechanisms. In addition, in vivo studies of ponatinib treatment alone or in combination with Akt/mTOR inhibitors will also provide further validation of the conclusion.

## 4. Materials and Methods

### 4.1. Cell Lines and Cell Culture

Human HCC cell line SK-Hep-1 was purchased from Cell Bank of Chinese Academy of Sciences (Shanghai, China) in October 2016. Human HCC cell line SNU-423 was purchased from American Type Culture Collection (ATCC, Manassas, VA, USA) in October 2016. They were cultured in Dulbecco’s Modified Eagle’s Medium (DMEM) (Hyclone, Logan, UT, USA) (SK-Hep-1) or Roswell Park Memorial Institute 1640 Medium (RPMI-1640) (Hyclone, Logan, UT, USA) (SNU-423) supplemented with 10% fetal bovine serum. All cells were incubated in a humiliated atmosphere with 5% CO_2_ at 37 °C. The culture medium was changed two to three times a week. The two cell lines were tested and authenticated using short tandem repeat (STR) matching analysis. The Cell Bank of Chinese Academy of Sciences (Shanghai, China) routinely performs single nucleotide polymorphism (SNP) and STR analysis to confirm cell line identity.

### 4.2. Materials and Antibodies

All inhibitors were purchased from Selleck Chemicals (Houston, TX), prepared as 10 mM stock solutions in DMSO, and further diluted with culture medium before use. Annexin V-FITC/PI apoptosis detection kit and cell cycle detection kit were purchased from KeyGen BioTECH (Nanjing, China). The 3-(4,5-dimethylthiazol-2-yl)-2,5-diphenyltetrazolium bromide (MTT), DMSO, and Hoechst 33342 were obtained from Solarbio (Beijing, China). Antibodies for Src/p-Src, Mek/p-Mek, Erk/p-Erk, PI3K/p-PI3K, PDK1/p-PDK1, AKT/p-AKT, mTOR/p-mTOR, PTEN/p-PTEN(S380/T382/383), JNK/p-JNK, c-Jun, p38/p-p38, JAK2/P-JAK2, Stat3/p-Stat3, Rb/p-Rb, CDK4, CDK6, Cyclin D1, CDK2, Cyclin E1, RAD51, Bcl-2, Bax, cleaved caspase-3, and cleaved caspase-7 were all purchased from Cell Signaling Technology (Danvers, MA, USA). Tubulin and GAPDH primary antibodies were purchased from ZSGB-Biotechnology (Beijing, China). Horseradish peroxidase (HRP)-conjugated secondary antibodies against rabbit and mouse IgG were obtained from BBI life sciences (Shanghai, China).

### 4.3. Cell Proliferation Assay

Cell viability was determined using the MTT assay. Cells (3–5 × 10^3^ cells/well) were seeded in 96-well flat-bottomed plates and cultured for 24 h. Then, the kinase inhibitors were diluted into the medium in a 2-fold dilution series (0.0012 to 20 μM) and added into the wells after the medium in the well was removed. After 72 h of treatment, 50 μL MTT solution (5 mg/mL) was added to each well after the medium was removed, and the cells were incubated for 4 h at 37 °C. Then, the medium was removed, and 200 μL DMSO was added to each well. Absorbance at 492 nm was measured using a BioTek microplate reader (Winooski, VT, USA). Half-maximal inhibitory concentration (IC_50_) values were estimated using SPSS 19.0 software. Each experiment was performed in triplicates and at least three times.

### 4.4. Colony Formation Assay

SK-Hep-1 and SNU-423 cells (500 cells) were plated in 6-well plates. After 24 h incubation, the cells were treated with various concentrations of ponatinib and then cultured for an additional 10 days. Then, the cells were stained with 0.5% crystal violet and photographed. The absorbance of stained cells at 595 nm was measured using a BioTek microplate reader (Winooski, VT, USA). The clone formation rate was calculated as follows: colony formation (% of control) = (ODtreated cells − ODblank)/(ODcontrol − ODblank) × 100%.

### 4.5. In-Vitro Drug Combination Study

To test if treatment with rapamycyn or temsirolimus would sensitize the HCC cells to ponatinib treatment, cells were treated with ponatinib, rapamycin, and temsirolimus, either alone or in combination with a series of drug concentrations. MTT assay was used to measure cell proliferation.

To evaluate the interaction between two drugs, the Chou-Talalay method was used to calculate the combination index [54]. The combination index (CI) = D1/D’1 + D2/D’2, in which D1 and D2 were the concentrations of drug 1 and drug 2 in the combination that achieved a certain percentage of inhibition, while D’1 and D’2 were concentrations of drug 1 or drug 2 alone to achieve the same level of inhibition.

### 4.6. Apoptosis Assay

The Annecin V-FITC/PI apoptosis detection kit was used for the apoptosis assay. For analysis of cell apoptosis, cells were treated with ponatinib for 36 h, washed twice with phosphate buffered saline (PBS), digested with 0.25% trypsin without ethylenediaminetetraacetic acid (EDTA), and centrifuged at 1000 rpm for 3 min. Then, the cells were harvested, and the concentration was adjusted to 1 × 10^6^ cells/mL. The cells were resuspended in 500 μL binding buffer and dyed with 5 μL Annecin V-FITC and 5 μL PI for 15 min at 37 °C. The apoptosis rate was measured with a BD FACSCanto II flow cytometer (BD Biosciences, San Jose, CA, USA). Experiments were performed three times for each group.

### 4.7. Cell Cycle Analysis

A cell cycle detection kit was used for the cell cycle analysis. Cells were treated with ponatinib for 36 h, collected, and fixed in 70% ethanol at 4 °C overnight. Following the manufacturer’s protocol, cells were washed twice with PBS and treated with RNase A and propidium iodide (PI) at 37 °C for 1 h. Then, the samples were measured using a flow cytometer.

### 4.8. Morphological Analysis by Hoechst Staining

Cells (1 × 10^5^ cells/well) were seeded in a 6-well plate for 24 h before treatment with various concentrations of ponatinib for 36 h. Then, the cells were washed three times with PBS and stained with Hoechst 33342 according to the manufacturer’s protocol. Nuclear morphology of cells was examined under a fluorescence microscopy (Olympus, 1X51, Tokyo, Japan).

### 4.9. Western Blot Analysis

After treatment with ponatinib for 36 h, cells were suspended in radio-immunoprecipitation assay (RIPA) cell lysis buffer and lysed by homogenization. The cell lysates were collected by centrifugation, and the protein concentration was quantified using the BCA protein quantitation kit (Boster Biotechnology, Wuhan, China). A total of 100 μg protein per sample was separated by SDS-PAGE and then transferred onto polyvinylidene fluoride (PVDF) membranes (Millipore, Billerica, MA, USA). The membranes were blocked using 5% skim milk at room temperature for 1 h and incubated at 4 °C overnight with primary antibodies in 5% skim milk. Primary antibodies were detected with a corresponding HRP-conjugated IgG antibody, and bands were visualized using an enhanced chemiluminescent (ECL) blot detection system (Transgene, Beijing, China). Image J software was used to quantify the grey value of the bands.

### 4.10. Statistical Analysis

Statistical analyses were carried out using the SPSS 19.0 statistical software (SPSS Inc., Chicago, IL, USA). Statistical significance was analyzed using Student’s *t*-test, and *p* < 0.05 was considered statistically significant; *, *p* < 0.05; **, *p* < 0.01; ***, *p* < 0.001.

## Figures and Tables

**Table 1:** Classification and function of nine protein tyrosine kinase (PTK) inhibitors used in this study.

Drugs	Molecular Weight (g/mol)	Target PTKs
Ponatinib	532.56	BCR-ABL, VEGFR, PDGFR, FGFR, EpH kinase, SRC-family protein-tyrosine kinases, and Kit, RET, TIE2, Flt-3 kinase inhibitor
Sunitinib	398.47	PDGFRα, PDGFRβ, VEGFR1, VEGFR2, VEGFR3, c-KIT, Flt-3, CSF-1R, RET tyrosine kinase inhibitor
Sorafenib	464.83	KIT, Flt-3, VEGFR-2, VEGFR-3, PDGFR-β tyrosine kinase, C-Raf, B-Raf and mutant B-Raf kinase inhibitor. Unique in targeting the Raf/Mek/Erk pathway.
Dasatinib	488.01	SRC family (SRC, LCK, YES, FYN) tyrosine kinase, BCR-ABL, c-Kit, EpHA2, PDGFRβ kinase inhibitor
Pazopanib	437.52	VEGFR1, VEGFR2, VEGFR3, PDGFRα, PDGFRβ, c-kit tyrosine kinase inhibitor
Bosutinib	530.45	Src-family (Src, Lyn, Hck) tyrosine kinases, BCR-ABL kinase inhibitor
Gefitinib	446.90	EGFR/Her1/ErbB-1 tyrosine kinase inhibitor
Afatinib	485.94	EGFR (ErbB1)/HER2 (ErbB2)/ HER4 (ErbB4) tyrosine kinase inhibitor
Midostaurin	570.64	PKCα/β/γ, Syk, Flk-1, Akt, PKA, c-Kit, c-Fgr, c-Src, Flt-3, PDFRβ and VEGFR1/2 tyrosine kinase inhibitor

The following criteria were used to select drugs for the study: (1) inhibitors that strongly inhibit some cells but not too many; (2) inhibitors that inhibit a significant number of cells at higher concentration, but the spectrum of cells are not too broad. An automatic screen was set up based on these two criteria. If an inhibitor inhibited more than one cell line but fewer than 20 cell lines with IC_50_ below 0.01 nM, the inhibitor scored one point. If a drug inhibited between two and 20 cell lines with IC_50_ below 0.1 nM, it scored one point. With the six-point screen, the scores were tallied. If a drug scored four or above, the inhibitor was automatically chosen. With these considerations, the nine PTK inhibitors in the table were chosen. The targeted PTKs of these inhibitors can be found in the two websites (http://www.drugbank.ca and http://www.rxlist.com).

**Table 2:** The IC_50_ (μmol/L) of different single agents in HCC cell lines.

	SK-Hep-1	SNU-423
Ponatinib (μmol/L)	0.288 ± 0.044	0.553 ± 0.041
Sunitinib (μmol/L)	0.568 ± 0.070	2.720 ± 0.036
Sorafenib (μmol/L)	3.896 ± 0.037	3.125 ± 0.034
Dasatinib (μmol/L)	0.902 ± 0.107	9.758 ± 0.044
Pazopanib(μmol/L)	3.951 ± 0.041	9.777 ± 0.030
Bosutinib (μmol/L)	0.840 ± 0.042	3.062 ± 0.041
Gefitinib (μmol/L)	5.557 ± 0.029	5.752 ± 0.048
Afatinib (μmol/L)	1.218 ± 0.031	2.400 ± 0.024
Midostaurin (μmol/L)	3.363 ± 0.053	1.640 ± 0.070

The IC_50_ values were determined from the data in Figure 1A. Individual IC_50_ results are the mean ± SD from at least three separate experiments.

**Table 3:** Dose-response profiles of ponatinib + temsirolimus and ponatinib + rapamycin drug combinations.

Parameter	SK-Hep-1	SNU-423
IC_50_-ponatinib (μM)	0.288 ± 0.044	0.553 ± 0.041
IC_50_-temsirolimus (μM)	0.341 ± 0.197	11.407 ± 0.104
IC_50_-rapamycin (μM)	1.718 ± 0.282	22.665 ± 0.097
IC_50_-ponatinib + temsirolimus (μM)	0.002 ± 0.001	0.066 ± 0.009
IC_50_-ponatinib + rapamycin (μM)	0.019 ± 0.009	0.042 ± 0.007
CI_50_-ponatinib + temsirolimus	0.013	0.122
CI_50_-ponatinib + rapamycin	0.077	0.078

In treatments, two drugs were used and mixed in a 1:1 molar ratio. The cell proliferation assay was performed using a serial dilution within the indicated concentration range.
